# P300 response among children and adolescents: Age and stimulus effects

**DOI:** 10.6026/973206300211332

**Published:** 2025-06-30

**Authors:** Sangeeta Gupta, Arun Prasad, Gaurav Gupta

**Affiliations:** 1 Department of Physiology, All India Institute of Medical Sciences (AIIMS) Gorakhpur, Uttar Pradesh, India; 2 Department of Paediatrics, All India Institute of Medical Sciences (AIIMS) Patna, Bihar, India; 3 Department of General Surgery, All India Institute of Medical Sciences (AIIMS) Gorakhpur, Uttar Pradesh, India

**Keywords:** P300, ERP, age, target stimulus, intensity, probability, latency, amplitude, children, adolescents

## Abstract

The association between age and P300 components and also the influence of stimulus-related variations, particularly intensity and probability in children and adolescents is of interest. P300 amplitude and latency and the effects of stimulus variation on P300 components were measured in 60 subjects in the age-group of 5-18 years and analysed with one-way ANOVA. P < 0.05 was considered as statistically significant. Mean P300 latency decreased and mean N2-P3 amplitudes increased with statistical significance (p<0.001) (one-way ANOVA) with substantially greater changes till 8-11 years of age. P300 latency varied inversely with stimulus intensity and probability while N2-P3 amplitude varied positively with stimulus intensity and adversely with change in the probability in all the age-groups (p<0.001) (one-way ANOVA). Age affects both P300 latency and amplitude in the children and adolescents in a characteristic pattern and stimulus variations affect the P300 responses relatively uniformly in different younger age-groups.

## Background:

Late childhood and adolescence is marked by significant changes in physical, cognitive, emotional and social behaviour [1]. Numerous tests have been developed to evaluate cognitive ability of the individuals. Event-related potentials (ERPs) are among the more direct ways to investigate the cognition by examining the brain responses while individuals perform cognitive tasks. The late components of the event-related potential (ERP), in particular the P300 is one of the most extensively researched and reliable electrophysiological variables. P300 has been used to index differences in modes of information processing and cognitive skill from childhood through adulthood [2]. P300 follows a specific trajectory across the lifespan reflecting brain maturation in childhood and adolescence and degenerative effects in older age [3]. Using an odd-ball paradigm P300 can be elicited by auditory or visual stimuli as a late cortical neurophysiological response. It is recorded as a large positive peak approximately 300 ms after the stimulus onset in the waveform. P300 ERP component has been reported to provide a neurophysiological index of cognitive dysfunctions [4]. Brain maturation plays a crucial role in eliciting the P300 response, especially in developing children which in turn is strongly influenced by physical and hormonal transitions in this period. The majority of the research has been conducted on adults and children, with a little amount of attention paid to adolescents [5]. Thus, it is necessary to comprehend the P300's properties in this group. To provide relevant information on impaired cognitive and neurophysiological function, as well as to measure the rate of mental decline over time, P300 measures require normative data to which populations of interest can be compared. Age-related changes in cognition as well as the ways in which the brain processes and processing resources used during cognitive tasks alter with age can also be revealed by normative investigations. It is mostly on adult populations, the P300 alterations during the lifespan have been the focus of the majority of normative research conducted to date [6, 7, 8-9]. The present study investigated changes in the latency and amplitude of P300 in late childhood and adolescence. Previous studies in the adult age-groups have demonstrated age effects on both the amplitude and latency of the P300 peak in terms of prolongation of the latency of P300 whereas diminution of the amplitude [7, 8-9]. On the other hand, findings on early developmental processes in the younger age-groups revealed decrease in P300 latency, whereas P300 amplitude are found to either increase during childhood or showed no change [10, 11, 12-13]. The outcomes of the studies that have investigated the relationship between P300 components and age in early to late adolescence have been conflicting [14, 15-16]. These inconsistent findings are most likely due to the variations among research in the age groups that were examined. Polich *et al.* (1990) also reported great within-subject variability in P300 amplitude in children, which could result in unreliable findings in studies with small sample sizes [10]. Moreover, the impact of stimulus variables on P300 components has not received much attention. The presumed endogenous rather than exogenous origin of P300 ERP component and hence considering it comparatively immune to the effects of stimulus elements, may be attributable to the limited research [17]. There are few studies which have investigated the role of stimulus factors employing the auditory stimuli and both P300 amplitude and latency have been found to be affected by variation in auditory stimulus factors [18, 19]. Target stimulus intensity and probability have been stated as the primary determinant of the P300 size and timing. P300 amplitude increased as stimulus intensity increased and decreased as stimulus probability increased. While similar analyses on P300 latency have shown that P300 latency decreased significantly as stimulus intensity increased and also decreased when target probability increased. However, this significant P300 amplitude and latency variability due to changes in stimulus parameters has been suggested to vary with the demographics of the subject population [18]. Even if the majority of the studies are now available from different countries, there is a significant void in the literature from Indian population. In order to close this gap, this study investigates the precise impacts of age and stimulus variations on P300 measurements in childhood and adolescence in the Indian settings. It seeks to accomplish this by offering important new perspectives on age-related cognitive dynamics in a group that is frequently under-represented in the corpus of existing research. Therefore, it is of interest to report P300 responses among children and adolescents and to examine the impact of age and stimulus-related variations on the responses in this age-group.

## Materials and Methods:

The study was conducted on 60 subjects in the age-group of 5-18 years at Neurophysiology laboratory, Department of Physiology, at a tertiary care institute for duration of six months (from August 2018 to February 2019). Subjects recruited were those who visited outpatient clinics with various acute illnesses or for a health examination unrelated to neurologic or psychiatric illness. Normal neuro-otological examination with no signs of cognitive or behavioural impairment was confirmed before inclusion. The study was approved by Institutional ethics committee (IEC Ref no. AIIMS/Pat/IEC/2018/281). Informed consent was obtained before undertaking the tests with minor assent forms/consent forms appropriately filled by the participants/parents. Considering more marked age-related changes and narrower age ranges recommendation in children and adolescents, participants were classified into four different cross-sectional samples that covered the following age groups: 5-7 years, 8-11 years, 12-14 years and 15-18 years [20]. Purposive sampling technique was used to acquire the data and 15 subjects comprised each age-group. All the subjects were ensured to be comparable with respect to the time of food intake in relation to the test, grade point average/academic performances and body temperature [21].

## Procedure:

The P300 test was performed on Neuro-MEPω EMG and EP digital neurophysiological system software in Neurophysiology laboratory, AIIMS, Patna. This was specialized software which allowed for the measurement and analysis of P300 responses to auditory stimuli. It was a single channel recording performed in a quiet environment. Skin preparation was done before the application of surface electrodes. The international 10-20 system was employed for electrode placement. Gold-plated disc electrodes were used. Ground at forehead (Fpz) and reference electrode was placed at right mastoid (M2). ERP waveforms were recorded in all the three active electrode locations (Fz, Cz and Pz) separately. The impedance was kept at less than 5 kω (kiloohms). Patients were properly trained and instructed to discriminate the two types (target and non-target/standard) of stimuli before starting the test. They were instructed to detect rare (meaningful/target) stimuli within a series of frequent (non-meaningful/standard/non-target) stimuli (2000/1000Hz). Stimuli with condensation polarity were delivered via headphones binaurally with an inter-stimulus interval of 1000 ms. Low and high stimulus intensities (45 and 85 dB SPL) with two target stimulus probability conditions (20 % and 50 %) in each participant were delivered (Table 1 - see PDF). Patients were instructed to keep their eyes open and to avoid eye movements. Electrical signals were filtered with 35 Hz high-cut and 0.5 Hz low-cut filters. A 700 ms time window was employed. Instructions were provided to the subjects to press a button when the target stimulus was detected and not to respond when the standard/non-target was presented. Two records of 100 trials for all sets of experiments were recorded and analysed [22]. Participants with 33% or higher false positive errors (button press on a non-target stimulus) or false negative errors (no button press on a target stimulus) were excluded from the dataset. P300 was measured as the maximal positive peak after N2 within the range of 250 to 700 milliseconds after stimulus presentation. The amplitude of P300 was measured from the peak of N2 to the peak of P300 (N2-P3). All the data recorded from Cz and Pz location were measured and analysed.

## Statistical analysis:

For aiding analysis and comparison, the collected data was organised in spread sheets using Microsoft Excel software. The data was expressed as mean ± standard deviation. P300 amplitude and latency among the four age groups were compared with one-way ANOVA (analysis of variance). Post hoc Tukey HSD test was used for multiple comparisons across age ranges. Pearson's correlation coefficient (r) was calculated between age and P300 variables. Effects of stimulus variation on P300 amplitude and latency were measured with one-way ANOVA. P < 0.05 was considered as statistically significant. The analyses were performed using the statistical software, Stata: version 12 (StataCorp LLC 4905 Lakeway Drive College Station, Texas 77845-4512 USA).

## Results and Discussion:

The study intended to examine the relationship between age and P300 components in the early developmental ages *i.e.* childhood and adolescence age group. The behaviour of P300 components with stimulus-related changes, particularly intensity and probability have also been sought for in the younger age-groups. The study group consisted of 60 participants aged 5-18 years (mean age ± SD: 11.9 ± 4.24 years), taking off P300 responses with high false positive and false negative error rates. Thirty participants (50%) were males and 30 (50%) were females. P300 latency and N2-P3 amplitudes were compared among the participants in four different age-group categories [5-7 years (group 1), 8-11 years (group 2), 12-14 years (group 3) and 15-18 years (group 4)] (each with fifteen participants) (Table 2 - see PDF). Mean values of P300 latency (ms±SD) (372.06±23.67 at Pz and 370.33±19.16 at Cz) in childhood age-group (5-7 years) decreased to 302.2±5.01 and 302.73±5.03 (15-18 years) (at Pz and Cz respectively). The difference was statistically significant (p< 0.001) (one-way ANOVA) (Table 2 - see PDF). However, majority of the latency reduction (up to 309±12.84 ms at Pz, 308.6±12.42 ms at Cz) was observed till 8-11 years age group (group 2). The decrease beyond that was not statistically significant (group 2-3, 2-4, 3-4 (p>0.05) (Tukey HSD, one-way ANOVA) (Table 2 - see PDF). P300 latency, hence, demonstrated a characteristic decline from the childhood age-group further. And, the majority of the latency reduction (up to 309±12.84 ms at Pz) was found to be evident till group 2 (8-11 years age group). It further decreased till the age of 18 years, but the difference was not statistically significant [(group 2-3, 2-4, 3-4 (p>0.05)] (Tukey HSD, one-way ANOVA) (Table 2 - see PDF). The findings hence indicated a greater decline till 11 years of age. Similar latency changes have been documented in previous studies with adolescents as participants [22, 23]. However, there has been a little inconsistency with the age when it attained stability. Katsanis *et al.* have stated that P300 latency continues to decrease until late adolescence with stability acquired through the early twenties [23]. However, other similar studies have reported this decrease to reach its limit at an earlier age (12-15 years) which is in line with our findings [13, 20]. These findings in children and adolescents are contrasting in relation to those in adulthood which showed a rather steady P3 latency which was succeeded by a slow age-related increase during adulthood in studies with adult participants [14, 15]. Our data observed a P300 latency of 372.06±23.67 ms (at Pz) in 5-7 years age-group which comply with previous reports [13]. However, the above age-group has also been found to show greater P300 latency values (400 to 500 ms) in many past researches [15, 24]. Variation in the methodologies used may be attributable for the observed differences. The P300 latency has been stated as an index for speed and efficiency of information processing in the brain. Rapid development in the cortical association areas has been suggested to be the cause of the decrease in latency observed during the adolescent years. Development of more efficient resource allocation in relation to time, with cognitive development process in the younger individuals is the likely explanation provided [16]. In a study by Brickman *et al.* (2012), white matter fiber tracts were found to continue developing until they reach a brief plateau, at about age 30, after which they begin degenerating [25]. And it was further inferred that myelination and P300 latency may be related. With regard to N2-P3 amplitudes, the comparison among the four age-groups was statistically significant (p<0.001) (one-way ANOVA) (Table 2 - see PDF). Amplitudes values (µv±SD) showed an increasing trend till group 3 with that of 8.04±1.81 (Pz) and 6.81±1.31 (Cz) in group 1, 10.5±1.8 (Pz) and 9.36±1.18 (Cz) in group 2 and 11.91±1.92 (Pz) and 10.76±1.72 (Cz) in group 3. Slightly decreased values (11.78±1.37 at Pz 10.71±1.78 at Cz) was noted in group 4. Multiple comparisons with groups 2-3, 2-4 and 3-4 revealed p-value >0.05, indicating that the major rise in the amplitude values occurred till group 2 (8-11 years). The findings are in line with previous studies [14, 24]. The P300 amplitude has been suggested to indicate neural power or cognitive resources that grow with age. Rapid increase in the information processing during early childhood has been stated as the likely cause of the increase in the P300 amplitudes [3].

Figure 1 (see PDF) depicts a representative P300 record for group 1 (5-7 years) (with increased P300 latency values as 363 ms while relatively lower N2-P3 amplitudes as 9.2 µv, while Figure 2 (see PDF) illustrates a record with remarkable latency decline and amplitude augmentation in group 3 (12-14 years). To examine an overall correlation of ERP P300 components (at Pz) with age and obtain the strength and direction of the relationship, Pearson correlation analysis was performed. A strong negative correlation between age and P300 latency (Pz) (r = - 0.78, r^2^=0.61, p<0.001) was revealed (Figure 3 - see PDF). While correlation with N2-P3 amplitudes (Pz) showed a moderate positive correlation (r = 0.7, r^2^=0.50, p<0.001) (Figure 4 - see PDF). The above findings indicated that as the age (years) increased (5 to 18), P300 latency decreased while N2-P3 amplitude increased with significant association. The comparison of P300 response between the age-groups was statistically significant for both at Cz and Pz electrode locations. N2-P3 amplitude recorded at midline parietal (Pz) location, however, was higher than those at Cz in all the age-groups: p=0.007, t=3.13 (5-7 years), p=0.03, t=2.44 (8-11 years), p=0.008, t=3.06 (12-14 years) and p=0.01, t=2.77 (15-18 years) (paired t-test). No significant variation in P300 latency values with respect to electrode locations in various age-groups noted (p>0.05) (paired t-test). Relatively higher N2-P3 amplitudes at midline parietal (Pz) location found in our study is consistent with the findings reported in the literature. Variations in cellular volume, skull density and width under the electrode sites and participants' physiological properties including corpus callosum anatomical features have been implicated in the above mentioned rise [26]. To analyze the effects of target stimulus intensity and probability which are the major determinants of P300 responses, P300 was recorded in four different experimental conditions with different sets of target stimulus intensity (SI) (45 and 85 dB SPL) and probability (SP) (20 % and 50 %) (Table 1 - see PDF, Table 3 - see PDF). The changes in P300 responses with the above-mentioned target stimulus variations in four different sets were compared separately in each age-group. Effects of target stimulus variation were evident in all the age-groups.

The comparison of P300 latency values (Pz) among the four experimental sets provided statistically significant results (p<0.001) (one-way ANOVA) (Table 3 - see PDF). P300 latency varied inversely with stimulus intensity and probability in different sets analysed. The variation within the various groups (sets) was significant (1-2, 1-3, 1-4, 2-4, 3-4) (p<0.001) (Tukey HSD, one-way ANOVA) (Table 3 - see PDF). However, no significant change was noted within set 2 and 3 (Set 2: SI: 85 dB SPL, SP: 20 %) (Set 3: SI: 45 dB SPL, SP: 50 %) (p>0.05). The comparison of N2-P3 amplitude values (Pz) among the four experimental sets provided statistically significant results (p<0.001) (one-way ANOVA) (Table 3 - see PDF). N2-P3 amplitude varied positively with stimulus intensity while change in probability adversely changed the amplitude values in the different sets analyzed. The variation within the various groups (sets) was significant (1-2, 1-3, 2-4, 3-4) (p<0.001) (Tukey HSD, one-way ANOVA) (Table 3 - see PDF). However, within group comparison for set 1 and 4 revealed no significant change (Set 1: SI: 45 dB SPL, SP: 20 %;) (Set 4: SI: 85 dB SPL, SP: 50 %) (P>0.05). Effect of target stimulus intensity was, hence, evident in all the sets of experiments, in the form of increased N2-P3 amplitude and decreased peak latency with increased SI. Shorter P300 latency at 85 dB SPL than 45 dB SPL reflects an easier discrimination and classification of the stimuli when the intensity increases [27]. Similarly, findings with larger P300 amplitude at 85 dB SPL than at 45 dB SPL are also in concordance with previous studies which obtained larger P300 amplitude with higher stimulus intensity [27, 28]. P300 amplitude represents the extent of the mobilization of attentional resources to process the stimuli presented during a task [28]. This, in turn, is influenced by arousal level and/or by the more intense stimuli with greater task significance [21]. This has been reported to result in larger amplitude of P300 component according to the triarchic model of Johnson (1986) [29]. Hence, larger amplitudes in response to increase in the stimulus intensity in the present study may be explained by an increase in attention and arousal provoked by the more intense stimulus.

Effect of another crucial determinant, the target stimulus probability demonstrated decreased N2-P3 amplitude as well as P300 latency with the increase in the former. The findings comply with previous similar studies [30, 31]. Additionally, P300 latency changes with probability had characteristic additive effects in the sets with increase in the stimulus intensity. P300 latencies decreased in both set 3 (SI: 45 dB SPL and SP: 50 %) and set 4 (SI: 85 dB SPL, SP: 50 %) as compared to set 1 (SI: 45 dB SPL and SP: 20 %), yet with a greater decline in set 4 (Table 3 - see PDF). With respect to amplitude changes, which are known to increase with the decrease in the probability levels, rise in both set 2 (SI: 85 dB SPL, SP: 20 %) and set 1 (SI: 45 dB SPL and SP: 20 %) was found when compared to set 3 (SI: 45 dB SPL and SP: 50 %), yet with a greater degree of rise in set 2, which delivered high intensity stimulus (Table 3 - see PDF). The above findings suggested additive effects of stimulus intensity in the sets. These findings are in line with the previous researches [29]. The effect of stimulus changes in terms of intensity and probability on P300 latency and amplitudes were similar for all the four -groups examined. The findings, hence, suggested that the P300 responses with respect to intensity or probability manipulations are not affected by cognitive changes during the maturation. Similar results were obtained in a study by Curry & Polich (1990) who included participants in the age-range of 8-19 years [31]. Another study with participants aged 5-19 years which reported P300 amplitude variations with the target stimulus probability to be similar across age groups, closely supports the present results [32].

## Limitations:

We did not examine the relationship of P300 latency with the reaction time which could have provided further insights into the decision process that invokes the response. P300 measures from Fz electrode location were not taken into consideration for analysis and comparison which would have extended the correlation findings reflected by a frontal P300 with age. Sex effects were not sought for in the study. The effect of stimulus intensity and probability could have been tested with greater variations in the ranges of intensity and probability.

## Conclusion:

P300 latency and amplitude exhibit significant age-related changes in the children and adolescents and the afore-mentioned findings seem to reflect developmental changes in mental processing in the younger individuals. Uniform influence of variation in target stimulus intensity and probability on P300 responses in different age-groups suggested that cognitive changes occurring during the maturation do not influence P300 components for the above manipulations. P300 component behaviour in the younger age-groups, however, needs further researches investigating the influence of other auditory attribute manipulation like frequency difference between the standard and target stimuli, stimulus sequence and stimulus modality to obtain better insights.

## References

[1] Blakemore S.J (2008). Nat Rev Neurosci..

[2] Friedman D (1989). Developmental Neuropsychology..

[3] van Dinteren R (2014). PLoS One..

[4] Sara G (1994). J Affect Disord..

[5] Polich J (1991). Electroencephalogr Clin Neurophysiol Suppl..

[6] Walhovd K.B (2008). Psychophysiology..

[7] Rossini P.M (2007). Prog Neurobiol..

[8] Ashford J.W (2011). J Alzheimers Dis..

[9] Kuba M (2012). Vision Res..

[10] Polich J (1990). Int J Psychophysiol..

[11] Sangal R.B (1998). Clin Electroencephalogr..

[12] Rozhkov V.P (2009). Neurosci Behav Physiol..

[13] Tsai M.L (2012). J Formos Med Assoc..

[14] Goodin D.S (1978). Electroencephalogr Clin Neurophysiol..

[15] Courchesne E (1978). Electroencephalogr Clin Neurophysiol..

[16] Brown W.S (1983). Electroencephalogr Clin Neurophysiol..

[17] Hillyard S.A, Kutas M (1983). Annu Rev Psychol..

[18] Polich J (1996). Int J Psychophysiol..

[19] Polich J (1987). Electroencephalogr Clin Neurophysiol..

[20] Picton T.W (2000). Psychophysiology..

[21] Polich J, Kok A (1995). Biol Psychol..

[22] Picton T.W (1992). J Clin Neurophysiol..

[23] Katsanis J (1996). Int J Psychophysiol..

[24] Martin L (1988). Electroencephalogr Clin Neurophysiol..

[25] Brickman A.M (2012). Neurobiol Aging..

[26] Ranjan R (2024). Hearing Balance and Communication..

[27] Sugg M.J, Polich J (1995). Biol Psychol..

[28] Lindín M (2005). Biol Psychol..

[29] Johnson R-Jr (1986). Psychophysiology..

[30] Polich J (1990). Int J Psychophysiol..

[31] Curry J.G, Polich J (1990). Developmental Neuropsychology..

[32] Ladish C, Polich J (1989). J Exp Child Psychol..

